# LC–MS/MS-based phytochemical profiling and preliminary in vitro anticancer-related bioactivity of *Euphorbia petrophila* ethanol extract

**DOI:** 10.1186/s12906-026-05470-8

**Published:** 2026-07-23

**Authors:** Mehmet Ali Güzel, İrem Nur Menevşe, Turgay Kolaç, Şengül Yüksel, Birol Mutlu

**Affiliations:** 1 İnönü University Vocational School of Health Services, Malatya, Turkey; 2 Department of Molecular Biology and Genetics, Inonu University, Malatya, Turkey; 3 Department of Medical Biology and Genetics, Faculty of Medicine, Inonu University, Malatya, Turkey; 4 Department of Biology, Faculty of Arts and Sciences, Inonu University, Division of Botany, Malatya, Turkey

**Keywords:** Apoptosis, Breast cancer, *Euphorbia petrophila*, LC–MS/MS, Lung cancer, Natural products, RT-qPCRf

## Abstract

**Background:**

Euphorbia species contain diverse secondary metabolites with reported biological activities, but *Euphorbia petrophila* remains pharmacologically underexplored. This study aimed to characterize the phytochemical profile of *E. petrophila* ethanol extract and evaluate its preliminary in vitro anticancer-related activity in lung and breast cancer cell models.

**Methods:**

Aerial parts of *E. petrophila* were extracted with ethanol using ultrasonic-assisted extraction. The extract was profiled by LC–MS/MS. Cytotoxicity was assessed by MTT assay in A549 and MDA-MB-231 cancer cells and in BEAS-2B and MCF10A non-tumorigenic epithelial cells. Apoptosis and cell-cycle distribution were analyzed by flow cytometry. Migration and invasion were evaluated using Transwell assays, and selected gene-expression changes were measured by RT-qPCR. Data were analyzed using appropriate analysis of variance and post hoc comparisons.

**Results:**

LC–MS/MS analysis identified phenolic acids and flavonoids, with gallic acid, quinic acid, ellagic acid, and isoquercitrin among the most abundant detected constituents. The extract reduced IC₅₀ value of in all tested cell lines, with the strongest cytotoxic effect observed in A549 cells. It increased apoptotic cell populations and promoted G0/G1 cell-cycle accumulation, particularly in A549 cells. The extract also markedly suppressed migration and invasion in A549 and MDA-MB-231 cells. These functional effects were accompanied by changes in TP53 and CDKN1A mRNA expression.

**Conclusions:**

*E. petrophila* ethanol extract shows preliminary antiproliferative, pro-apoptotic, anti-migratory, and anti-invasive activity in selected cancer cell models. However, because the findings are based on crude extract testing and transcript-level analyses, the results should be interpreted as preliminary and correlative. Further work should include extract standardization, LC–MS/MS method validation, broader normal-cell toxicity profiling, active-compound isolation, protein-level mechanistic assays, and in vivo safety and efficacy studies.

**Supplementary Information:**

The online version contains supplementary material available at https://doi.org/10.1186/s12906-026-05470-8.

## Introduction

Cancer represents a leading cause of mortality globally, characterized by uncontrolled cellular proliferation, genetic instability, and metastatic potential [1]. Breast cancer is the most frequently diagnosed malignancy among women, with an estimated 2.3 million new cases and over 670,000 deaths reported in 2022 alone [2]. Likewise, lung cancer remains the foremost cause of cancer-related deaths worldwide. Among its histological subtypes, adenocarcinoma accounts for approximately 40% of all cases, whereas non-small cell lung cancer (NSCLC) constitutes nearly 85% [3].

Conventional treatment modalities such as chemotherapy, radiotherapy, and immunotherapy are often associated with severe side effects due to their nonspecific cytotoxic effects on healthy tissues [4]. Consequently, the demand for safer and more selective cytotoxic agents, especially those derived from natural sources has grown considerably in recent years. In this context, medicinal plants offer a promising reservoir of bioactive compounds with potential applications in oncology [5].

The genus *Euphorbia* (family: Euphorbiaceae), consisting of over 2,000 species distributed across temperate and tropical zones, has been traditionally used to treat a wide spectrum of diseases, including inflammatory disorders, infections, and skin conditions [6]. Members of this genus exhibit vast morphological diversity, ranging from herbaceous plants and shrubs to succulent and cactus-like forms [7]. Pharmacological studies have confirmed the presence of diterpenoids, triterpenoids, flavonoids, and polyphenols in various *Euphorbia* species, many of which exhibit cytotoxic, antioxidant, anti-inflammatory, and antimicrobial effects [8].

Numerous *Euphorbia* species have demonstrated notable anticancer activities. For instance, euphol, isolated from *Euphorbia tirucalli*, showed dose-dependent cytotoxicity in 73 human cancer cell lines and significantly suppressed lung metastasis in mouse melanoma models [9]. *Euphorbia helioscopia* was associated with increased apoptotic cell populations and cell cycle arrest in multiple cancer cell types, while compounds from *Euphorbia kansui* and *E. fischeriana* were reported to enhance reactive oxygen species (ROS) production, modulate mitochondrial membrane potential, and downregulate anti-apoptotic proteins such as Bcl-2 [10–12].

Other promising species include *E. hirta*, which demonstrated caspase-mediated apoptosis in breast cancer cells (MCF-7) [13]. and *E. humifusa*, which inhibited TNF-α and MMP-9 expression in metastatic breast cancer models through NF-κB signaling suppression [14]. Moreover, extracts from *E. petiolata* and *E. hebecarpa* were shown to increase IFN-γ production and promote lymphocyte proliferation, suggesting immunomodulatory effects with potential relevance in cancer immunotherapy [15].

Despite these promising findings, *E. petrophila* remains poorly studied. The present study therefore evaluated the preliminary in vitro anticancer-related activity of *E. petrophila* ethanol extract against human lung and breast cancer cell models, including analyses of cell viability, apoptosis, cell-cycle distribution, migration, invasion, and selected gene-expression changes.

Overall, this work was designed to generate preliminary in vitro evidence on the biological activity of *E. petrophila* ethanol extract and to identify key experimental gaps that should be addressed before any therapeutic interpretation can be made.

## Material and methods

### Plant material and extraction procedure

Specimens of *E. petrophila* were collected from the Yama Mountain region in Hekimhan, Malatya (Eastern Anatolia, Turkey). The collected plant material consisted of the aerial parts of the plant, which were carefully cleaned and air-dried in a cool, dry, and shaded environment, protected from direct sunlight. The dried plant samples were stored in a cool, dry place until further use. The herbarium specimen details of *E. petrophila* are provided in Table 1. The plant species identification was performed by Prof. Dr. Birol MUTLU, a faculty member of the Department of Biology, Faculty of Science and Arts, İnönü University.

**Table 1:** Herbarium sample data of *E. petrophila* plant

Herbarium No	Plant species name	Collection time	Location	Elevation
16,549	*E. petrophila*	31/07/2023	B7; Malatya: Hekimhan, Yama Mountain	2350 m

For extraction, a 20 g sample of the powdered aerial parts of *E. petrophila* was subjected to solvent-assisted ultrasonic extraction. The aerial material was soaked in 150 mL of 96% ethanol for 2 h in an ultrasonic bath. The same process was repeated twice with 100 mL of ethanol for 30 min each. The resulting extract was filtered through Whatman No. 1 filter paper, and the pooled filtrate was concentrated under reduced pressure using a rotary evaporator to obtain the ethanolic extract. This process yielded 1.559 g of dry extract, representing an extraction yield of approximately 7.8% (w/w). The dried extract was stored at 4 °C in amber glass vials in a refrigerator until use. All experimental studies were conducted using this ethanol extract.

### LC–MS/MS analysis

The phytochemical constituents of the *E. petrophila* ethanol extract were characterized using a Thermo Scientific UHPLC system coupled with a TSQ Quantum Access MAX triple quadrupole mass spectrometer, equipped with an electrospray ionization (ESI) source. Chromatographic separation was achieved on a Thermo Accucore C18 column (100 mm × 2.1 mm, 2.6 µm) maintained at 30 °C. The mobile phase consisted of water with 0.1% formic acid (solvent A) and ethanol with 0.1% formic acid (solvent B), applied under a linear gradient program. The elution protocol was as follows: 0–2 min, 5% B; 2–10 min, linear ramp to 95% B; 10–12 min, held at 95% B; 12–13 min, return to 5% B; 13–15 min, re-equilibration. The flow rate was set at 0.3 mL/min with an injection volume of 10 µL. MS detection was conducted in negative ion mode with the following parameters: spray voltage 3500 V, capillary temperature 320 °C, sheath gas 35 arb, auxiliary gas 10 arb, and collision gas pressure 1.5 mTorr.Compound identification and quantification were performed in Multiple Reaction Monitoring (MRM) mode by comparing retention times, precursor/product ion transitions, and fragmentation patterns with authentic standards and literature-reported values. Prior to injection, extract samples were diluted to 1 mg/mL in LC–MS-grade ethanol and filtered through a 0.22 µm PTFE syringe filter [16].

### Cell lines and culture conditions

MDA-MB-231 (human breast adenocarcinoma, MCF10A (non-tumorigenic human mammary epithelial cells), A549 (human lung adenocarcinoma), and BEAS-2B (non-tumorigenic human bronchial epithelial cells) were procured from the American Type Culture Collection (ATCC, Manassas, VA, USA). All four cell lines were cultured in DMEM medium, supplemented with 10% (v/v) fetal bovine serum (FBS) and 1% (v/v) penicillin–streptomycin solution, and maintained under standard conditions in a humidified atmosphere at 37 °C with 5% CO₂.In parallel, the non-tumorigenic human mammary epithelial cell line MCF10A was cultured in DMEM/F12 (1:1) basal medium, supplemented with 5% (v/v) horse serum, 20 ng/mL epidermal growth factor (EGF), 0.5 μg/mL hydrocortisone, 100 ng/mL cholera toxin, and 10 μg/mL human insulin. All media and supplements were obtained from certified commercial suppliers and were prepared under sterile conditions. Cells were routinely passaged at ~ 80% confluence using 0.25% trypsin–EDTA and were confirmed to be mycoplasma-free prior to experimentation. These standardized conditions ensured reproducibility and physiological relevance across all downstream in vitro assays [17].

### IC₅₀ value of analysis using the MTT assay

The anticancer potential of the plant samples was evaluated in accordance with the protocol adapted from Sharma et al. (2019) using four human cell lines: BEAS-2B (non-tumorigenic lung epithelial cells), A549 (lung adenocarcinoma), MCF10A (non-tumorigenic mammary epithelial cells), and MDA-MB-231 (breast adenocarcinoma). Cisplatin was used as a positive control. All cell lines were cultured in DMEM medium supplemented with 10% fetal bovine serum (FBS) and 1% penicillin–streptomycin, maintained at 37 °C in a humidified atmosphere with 5% CO₂. Once cells reached 70–80% confluence, the medium was removed, and cells were washed with sterile PBS (pH 7.4). Trypsin–EDTA was added to detach cells, followed by neutralization with fresh complete medium. The suspension was centrifuged at 1000 rpm for 7 min, and the pellet was resuspended in fresh medium. Cells were then counted and diluted to a final density of 1 × 10^5^ cells/mL, and 1 × 10^4^ cells per well were seeded into 96-well plates. Plates were incubated for 24 h to allow cell adherence. Plant extracts were prepared in complete medium at final concentrations ranging from 0.8 to 800 µg/mL, and 100 µL of the test solution was added to each well after aspirating the old medium. Cells were incubated for another 24 h at 37 °C, 5% CO₂. Following treatment, the medium was aspirated, and 10 µL of MTT solution (5 mg/mL) and 90 µL of fresh medium were added to each well to achieve a final concentration of 0.5 mg/mL MTT.

Plates were incubated for 4 h, and absorbance was measured at 570 nm and 630 nm using an ELISA reader. IC₅₀ value of was calculated using the formula:

[(570 nm − 630 nm)_treated/(570 nm − 630 nm)_control] × 100. The IC₅₀ values were derived based on a logarithmic dose–response curve generated from the IC₅₀ value of data [18, 19].

### RNA isolation and RT-qPCR analysis

After 24 h of treatment with *E. petrophila* extract and cisplatin, cells grown in 6-well plates were harvested by trypsinization and centrifuged at 1000 rpm for 7 min. Total RNA was extracted from the resulting pellets using the PureLink™ RNA Mini Kit (Invitrogen™) following the manufacturer’s instructions. RNA quantity and purity were assessed with a Denovix DS-11 FX + spectrophotometer, and samples with an A260/A280 ratio of 1.8–2.0 were used for cDNA synthesis. Complementary DNA (cDNA) was synthesized from 1 μg of total RNA using the ProtoScript® First Strand cDNA Synthesis Kit. RT-qPCR was carried out on a StepOne™ Plus Real-Time PCR System (Applied Biosystems) using GoTaq® qPCR Master Mix (Promega). Each 20 μL reaction contained 100 ng cDNA, 200 nM forward and reverse primers, and nuclease-free water [20].

Thermal cycling conditions were:

- 95 °C for 10 min,
- Followed by 40 cycles of 95 °C for 10 s and 60 °C for 30 s.

Gene expression levels were normalized to GAPDH and calculated using the 2^ − ΔΔCt method. The primer sequences of the target genes used in this study are listed in Table 2. All RT-qPCR reactions were performed in biological triplicates and technical duplicates/triplicates All RT-qPCR reactions were performed in biological triplicates and technical duplicates/triplicates. Primer specificity was confirmed by melting curve analysis, and amplification efficiencies were calculated before relative quantification using the 2 − ΔΔCt method.

**Table 2:** Primer sequences used for RT-qPCR analysis

Gene	Forward primer (5′–3′)	Reverse primer (5′–3′)
TP53	CAGCACATGACGGAGGTTGT	TCATCCAAATACTCCACACGC
P21	GCCCAGTGGACAGCGAGCAG	GCCGGCGTTTGGAGTGGTAGA
BRCA1	TCGTGGTAACGGAAAGCGC	AAATCTCAACGAACTCACGCC
ATM	ATGAGTCTAGTACTTAATGATCTGCTTATCTG	TCTTCAGGTTGTAATTATATTTGTGGT
CDK4	ATGGCTACCTCTCGATATGAGC	CATTGGGGACTCTCACACTCT
CyclinD1	GCTGCGAAGTGGAAACCATC	CCTCCTTCTGCACACATTTGA

### Cell cycle analysis by flow cytometry

Cells were seeded into 6-well plates and treated with *E. petrophila* extract and cisplatin for 24 h. Following treatment, the culture media were aspirated, and each well was washed three times with PBS. Cells were detached by adding 500 μL of 0.25% trypsin and incubating at 37 °C for 5 min. After detachment, 1 mL of fresh medium was added to each well, and cell suspensions were transferred into sterile microcentrifuge tubes. Cells were gently resuspended and fixed by adding 70% ethanol (Sigma) and incubated at 4 °C for 2 h. After fixation, cells were centrifuged for 7 min, washed twice with PBS, and incubated with 0.1 mg/mL RNase A in 500 μL PBS at 37 °C for 30 min to degrade RNA. Subsequently, cells were stained with 0.05 mg/mL propidium iodide (PI) and incubated in the dark at 4 °C for 30 min. PI-stained cells were analyzed using a BD Accuri™ C6 Plus flow cytometer (BD Life Sciences). A minimum of 10,000 events per sample were acquired, and cell debris was excluded based on forward scatter (FSC) and side scatter (SSC) parameters. PI was excited with a 488 nm blue laser, and emission was detected using the FL2 channel (585/40 nm bandpass filter). Data were processed using BD Accuri™ C6 Plus software, and the distribution of cells across G0/G1, S, and G2/M phases was quantified. The impact of *E. petrophila* extract on the cell cycle was evaluated by comparing the phase distributions between treated and untreated groups of the same cell line [21].

### Apoptosis analysis by Annexin V-FITC/PI staining

Cells were seeded into 6-well plates and treated with *E. petrophila* extract and cisplatin for 24 h. After treatment, media were removed, and wells were washed three times with PBS. Adherent cells were detached using 500 μL of 0.25% trypsin per well and incubated for 5 min at 37 °C. The detached cells were collected by adding 1 mL of fresh medium and transferred into sterile microcentrifuge tubes. Following centrifugation at 1000 rpm for 7 min, each cell pellet was resuspended in 100 μL of Annexin V binding buffer (provided in the kit). To each sample, 5 μL of Annexin V-FITC conjugate (90 µg/mL) and 10 μL of propidium iodide (PI; 0.5 mg/mL) were added. Samples were gently vortexed and incubated for 15 min in the dark at room temperature. After incubation, 400 μL of additional binding buffer was added to each tube prior to flow cytometric analysis. Apoptotic profiles were analyzed using a BD Accuri™ C6 Plus Flow Cytometer (BD Life Sciences). A minimum of 10,000 events per sample were acquired. Debris was excluded based on forward (FSC) and side scatter (SSC) signals. Fluorescence was detected using a 488 nm blue laser:

- Annexin V-FITC was detected in FL1 channel (533/30 nm)
- PI was detected in FL2 channel (585/40 nm)

Data acquisition and quadrant-based analysis of early/late apoptosis were performed using BD Accuri™ C6 Plus software. The pro-apoptotic effect of *E. petrophila* extract was evaluated by comparing the percentages of apoptotic cells (Annexin V⁺/PI⁻ and Annexin V⁺/PI⁺) across different treatment groups of the same cell line [22].

### Migration and invasion assays using a transwell system

Cell migration and invasion abilities were assessed using the Boyden chamber (Transwell® system) with 8 μm pore-size polycarbonate membrane inserts (Transwell® Permeable Supports, Polycarbonate Membrane, Cat No: 3428, Corning Inc.). For migration assays, cells were seeded directly into the upper chamber without any matrix coating. For invasion assays, the upper surface of the membranes was coated with 1 mL of Matrigel® Basement Membrane Matrix (200–300 μg/mL, Corning®, Cat No: 354234) and incubated at 37 °C for 30 min to allow gel polymerization prior to seeding. In both assays, the Transwell inserts were placed into wells containing complete medium (chemoattractant) in the lower chamber, while cells suspended in serum-free medium were seeded in the upper chamber. After the incubation period (typically 24 h), non-migrated/invaded cells on the upper surface of the membrane were gently removed with a cotton swab. Cells that had traversed the membrane were fixed, stained, and analyzed. Quantitative assessment was performed by flow cytometry using the BD Accuri™ C6 Plus system, and the data were processed using BD Accuri™ C6 Plus software. Migrated and invaded cell populations were compared across experimental groups to evaluate the effects of *E. petrophila* extract [23].

### Statistical analysis

All experiments were performed in triplicate, and the results are expressed as mean ± standard deviation (SD). Statistical analyses were conducted to assess the significance of differences between groups. For in vitro cytotoxicity data obtained from multiple treatment groups across different cell lines, two way analysis of variance (ANOVA) followed by Tukey’s post hoc test was used to determine significant differences between treatments. Prior to ANOVA, data normality was assessed using the Shapiro–Wilk test, and homogeneity of variances was confirmed via Levene’s test. Statistical significance was set at *p* < 0.05. Experiments were performed as three independent biological replicates, each including technical triplicates.

## Results

### LC–MS/MS analysis

LC–MS/MS analysis revealed the presence of more than 30 distinct phenolic compounds in the *E. petrophila* extract. The identified metabolites predominantly included hydroxybenzoic acid derivatives, hydroxycinnamic acids, and flavonoid aglycones. Detailed quantitative data are presented in Table 3. Among the compounds detected, gallic acid (13,735.17 ng/mL), ellagic acid (9,490.81 ng/mL), quinic acid (12,012.05 ng/mL), and isoquercitrin (6,159.16 ng/mL) were found to be the most abundant constituents. These bioactive compounds are well-documented for their antioxidant, anti-inflammatory, and anticancer properties, and their high concentrations may contribute to the extract’s observed biological effects.

**Table 3:** Chemical composition of *E. petrophila* and validation parameters for LC–MS/MS-based quantification of phytochemical standard

		RT	Parent Ion (m/z)	Fragment Ions (m/z)	R^2^	LOD (ng/mL)	LOQ (ng/mL)	Recovery	µg/g dry extract
1	Hydroxybenzoic acid	7.82	137.02442	93.03471	0.9921	1.03	3.44	116.03	114.46 ± 8.36
2	Salicylic acid	10.93	137.02442	93.03468	0.9946	1.16	3.87	115.44	218.48 ± 8.09
3	Syringic acid	8.9	197.04555	197.04555	0.9949	1.06	3.54	111.27	74.60 ± 5.10
4	Gallic acid	3.7	169.01425	125.02461	0.9903	2.94	9.78	109.36	13,735.17 ± 862.87
5	Protocatechuic acid	6.24	153.01933	109.02949	0.9908	0.73	2.44	80.85	416.23 ± 18.75
6	Protocatechualdehyde	7.29	137.02442	136.01671	0.9909	0.46	1.55	71.73	1.73 ± 0.08
7	β-resorcylic acid	8.42	153.01933	67.01888	0.9935	0.84	2.79	112.81	99.82 ± 3.54
8	Gentisic acid	7.4	153.01933	153.01933	0.9913	0.51	1.7	104.55	464.24 ± 11.55
9	Coumaric acid	9.8	163.04007	119.05027	0.9928	1.01	3.37	98.47	46.31 ± 2.21
10	Ferulic acid	10.05	193.05063	134.03751	0.9933	1.39	4.63	102.84	544.05 ± 2.91
11	Sinapic acid	10.02	223.0612	193.01436	0.9934	1.03	3.44	98.72	16.44 ± 1.85
12	Chlorogenic acid	8.07	353.08781	191.05624	0.9976	0.8	2.65	105.09	608.96 ± 6.50
13	Quinic acid	0.93	191.05611	85.02962	0.9939	0.8	2.68	105.72	12,012.05 ± 600.26
14	Apigenin	13.24	269.04555	117.03464	0.9932	1.05	3.51	104.9	3.34 ± 0.10
15	Rhoifolin	10.12	577.15631	269.04552	0.9942	0.85	2.83	102.41	3.04 ± 0.05
16	Rutin hydrate	10.67	609.14611	300.02777	0.9912	1.28	4.28	100.29	1068.55 ± 18.37
17	Luteolin	12.53	285.04046	175.04039	0.9932	1.54	5.12	97.96	76.70 ± 2.58
18	Diosmetin	13.45	299.05612	284.03264	0.9821	1.24	4.13	98.75	87.69 ± 2.01
19	Quercetin	12.15	301.03538	151.00223	0.9955	0.62	2.07	104.12	1534.03 ± 40.50
20	İsoquercitrin	10.86	463.08820	301.03530	0.9890	1.09	3.63	101.24	6159.16 ± 22.04
21	Narcissin	11.60	623.16176	315.05100	0.9962	1.15	3.83	99.65	116.80 ± 4.02
22	Quercetin 3-rutinoside	11.43	447.09296	301.03530	0.9973	0.88	2.93	103.45	2577.26 ± 32.45
23	Isorhamnetin	13.21	315.05103	300.0278	0.9958	0.59	1.95	96.24	112.60 ± 2.11
24	Kaempferol	13.08	285.04046	136.01686	0.9924	0.57	1.91	96.28	44.44 ± 2.16
25	Afzelin	11.98	431.09837	285.04028	0.9951	1.96	6.53	101.28	157.81 ± 6.69
26	Nicotiflorin	11.31	593.15119	285.04041	0.9915	0.75	2.49	96.14	65.16 ± 2.67
27	Myricetin	11.2	317.0303	151.0022	0.9938	0.91	3.03	101.55	666.42 ± 7.16
28	Naringenin	12.34	271.0612	119.05035	0.9932	1.46	4.85	103.27	549.92 ± 17.77
29	Ellagic acid	10.93	300.99899	300.99872	0.9943	0.68	2.27	106.41	9490.81 ± 48.61

*RT* Retention time, *LOD* Limit of detection, *LOQ* Limit of quantification, *R*^*2*^ Correlation coefficient

### IC₅₀ value of assay using MTT

The antiproliferative effects of *E. petrophila* extract on cancerous cell lines (A549 and MDA-MB-231) and non-tumorigenic epithelial cell lines (BEAS-2B and MCF10A) were assessed using the MTT assay. Cells were treated with varying concentrations of the extract and cisplatin ranging from 0.8 to 800 μg/mL for 24 h. IC₅₀ value of percentages were calculated spectrophotometrically using absorbance values obtained at 570 and 630 nm. IC₅₀ values were then determined from logarithmic concentration–response curves based on the calculated IC₅₀ value of percentages.

Table 4 presents the IC₅₀ values of *E. petrophila* extract and cisplatin in different cell lines. In BEAS-2B cells, the IC₅₀ value of *E. petrophila* extract was 55.52 ± 2.61 μg/mL, whereas cisplatin showed an IC₅₀ value of 31.09 ± 0.98 μg/mL. In A549 cells, the extract exhibited an IC₅₀ value of 26.19 ± 1.19 μg/mL, while cisplatin showed an IC₅₀ value of 58.23 ± 2.12 μg/mL. In MCF10A cells, the IC₅₀ values were 43.99 ± 1.92 μg/mL for *E. petrophila* extract and 36.66 ± 1.23 μg/mL for cisplatin. Finally, in MDA-MB-231 cells, the IC₅₀ value of *E. petrophila* extract was 62.19 ± 3.10 μg/mL, whereas cisplatin showed an IC₅₀ value of 27.31 ± 0.74 μg/mL.

**Table 4:** IC₅₀ values of *E. petrophila* extract and cisplatin in different cell lines determined by MTT assay

Cell group	Cell type	*E. petrophila* extract IC₅₀ (μg/mL, mean ± SD)	Cisplatin IC₅₀ (μg/mL, mean ± SD	Difference
BEAS-2B	Non-tumorigenic bronchial epithelial cells	55.52 ± 2.61	31.09 ± 0.98	24.43
A549	Lung adenocarcinoma cells	26.19 ± 1.19	58.23 ± 2.12	−32.04
MCF10A	Non-tumorigenic mammary epithelial cells	43.99 ± 1.92	36.66 ± 1.23	7.33
MDA-MB-231	Triple-negative breast cancer cells	62.19 ± 3.10	27.31 ± 0.74	35.09

The selectivity index (SI) was calculated to evaluate the preferential cytotoxicity of *E. petrophila* extract toward cancer cells compared with non-tumorigenic epithelial cells. SI values were calculated using IC₅₀ values obtained from normal and cancer cell lines according to the following formula:

SI = IC₅₀ of non-tumorigenic cells/IC₅₀ of cancer cells

*SD* Standard deviation, *IC₅₀* half-maximal inhibitory concentration, *E. p.*: *E. petrophila*

These results indicate that *E. petrophila* extract showed the strongest antiproliferative effect against A549 cells among the tested cell lines. However, its effect was weaker than cisplatin in BEAS-2B, MCF10A, and MDA-MB-231 cells under the current experimental conditions.

An SI value greater than 2 was considered indicative of selective cytotoxic activity toward cancer cells. The findings obtained from the analysis are shown in Table 5.

**Table 5:** Selectivity index values of *E. petrophila* extract calculated from IC₅₀ ratios between non-tumorigenic epithelial cells and cancer cell lines

Comparison	Calculation	Selectivity Index
BEAS-2B vs A549	55.52/26.19	2.12
MCF10A vs MDA-MB-231	43.99/62.19	0.71

The SI value for BEAS-2B versus A549 was 2.12, suggesting selective cytotoxicity of *E. petrophila* extract toward A549 lung cancer cells compared with BEAS-2B non-tumorigenic bronchial epithelial cells. In contrast, the SI value for MCF10A versus MDA-MB-231 was 0.71, indicating that the extract did not show selective cytotoxicity toward MDA-MB-231 breast cancer cells compared with MCF10A non-tumorigenic mammary epithelial cells under the tested conditions.

### Apoptotic effects of *E. petrophila* extract

**Fig. 1:**
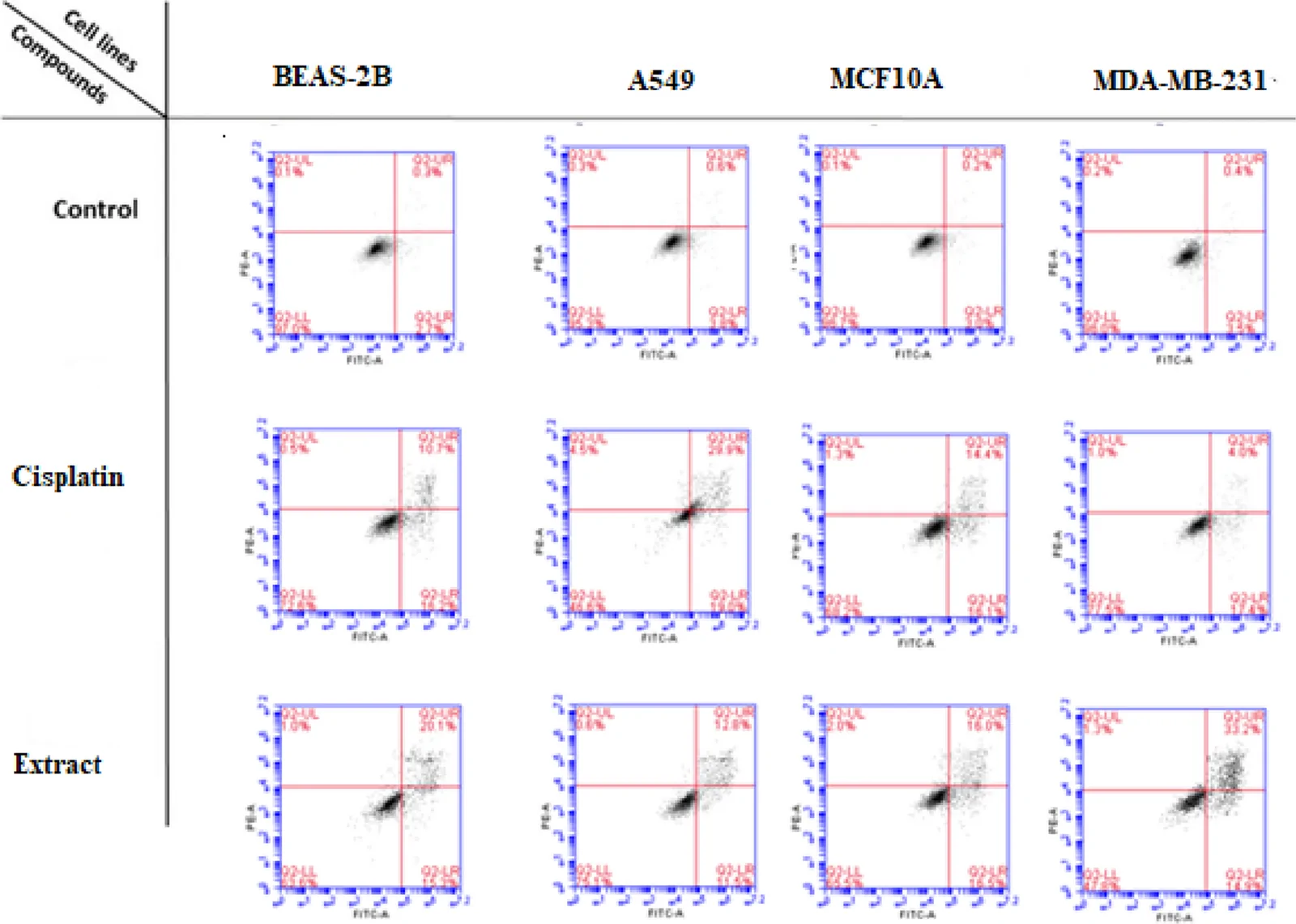
Flow cytometry plots showing apoptosis results in BEAS-2B, A549, MCF10A and MDA-MB-231cell lines following *E. petrophila* extract treatment

To evaluate whether the *E. petrophila* extract induces apoptosis in different cell types, flow cytometry-based apoptosis assays were performed on BEAS-2B (non-tumorigenic lung epithelial), A549 (lung adenocarcinoma), MCF10A (non-tumorigenic breast epithelial), and MDA-MB-231 (triple-negative breast cancer) cell lines. Cells were treated with 25 μg/mL of the extract for 24 h and stained with Annexin V-FITC and propidium iodide (PI), followed by analysis using a BD Accuri™ C6 Plus flow cytometer. Two-dimensional dot plots were generated displaying PI versus Annexin V-FITC fluorescence, and the data were interpreted based on quadrant distribution: Q3 (FITC −/PI −): Viable cells, Q1 (FITC +/PI −): Early apoptotic cells, Q2 (FITC +/PI +): Late apoptotic/secondary necrotic cells, Q4 (FITC −/PI +): Necrotic cells. The apoptosis rates of *E. petrophila* extract and cisplatin were evaluated in different cell groups. In the BEAS-2B cells, *E. petrophila* extract induced an apoptosis rate of 26.53 ± 1.02%, whereas cisplatin treatment resulted in a rate of 34.61 ± 2.03%, with a significant mean difference of −8.8% (*p* < 0.05). A549 cells exhibited a higher apoptosis rate with *E. petrophila* extract (49.06 ± 2.05%) compared to cisplatin (23.43 ± 1.36%), with a mean difference of 25.63%, which was statistically significant (*p* < 0.05). In MCF10A cells, the apoptosis rates were similar between *E. petrophila* extract (29.52 ± 1.21%) and cisplatin (31.96 ± 2.14%), with a mean difference of-2.44%, showing no significant difference (*p* > 0.05). Finally, in MDA-MB-231 cells, *E. petrophila* extract induced an apoptosis rate of 21.17 ± 0.25%, while cisplatin treatment resulted in 47.93 ± 3.15%, with a significant mean difference of-20.76% (*p* < 0.05). The flow cytometry images of the analyzed samples are shown in Fig. 1, and the amounts of apoptosis observed after the analysis are table presented in Table 6.

**Table 6:** The effects of *E. petrophila* extract and cisplatin treatment on apoptosis in different cell lines(IC50: μg/mL

Cell group	*E. petrophila* extract (% mean ± SD)	Cisplatin (% mean ± SD)	Mean Difference (Δ%)	Significance
BEAS-2B	26.53 ± 1.02	34.61 ± 2.03	−8.8	*p* < *0.05*
A549	49.06 ± 2.05	23.43 ± 1.36	25.63	*p* < *0.05*
MCF10A	29.52 ± 1.21	31.96 ± 2.14	−2.44	*p* > *0.05*
MDA-MB-231	21.17 ± 0.25	47.93 ± 3.15	−20.76	*p* < *0.05*

*S.D* Standard deviation, *E.p.A.O* The apoptosis rate of *E. petrophila* extract, *A.O* Apoptosis rate

### Cell-cycle results

**Fig. 2:**
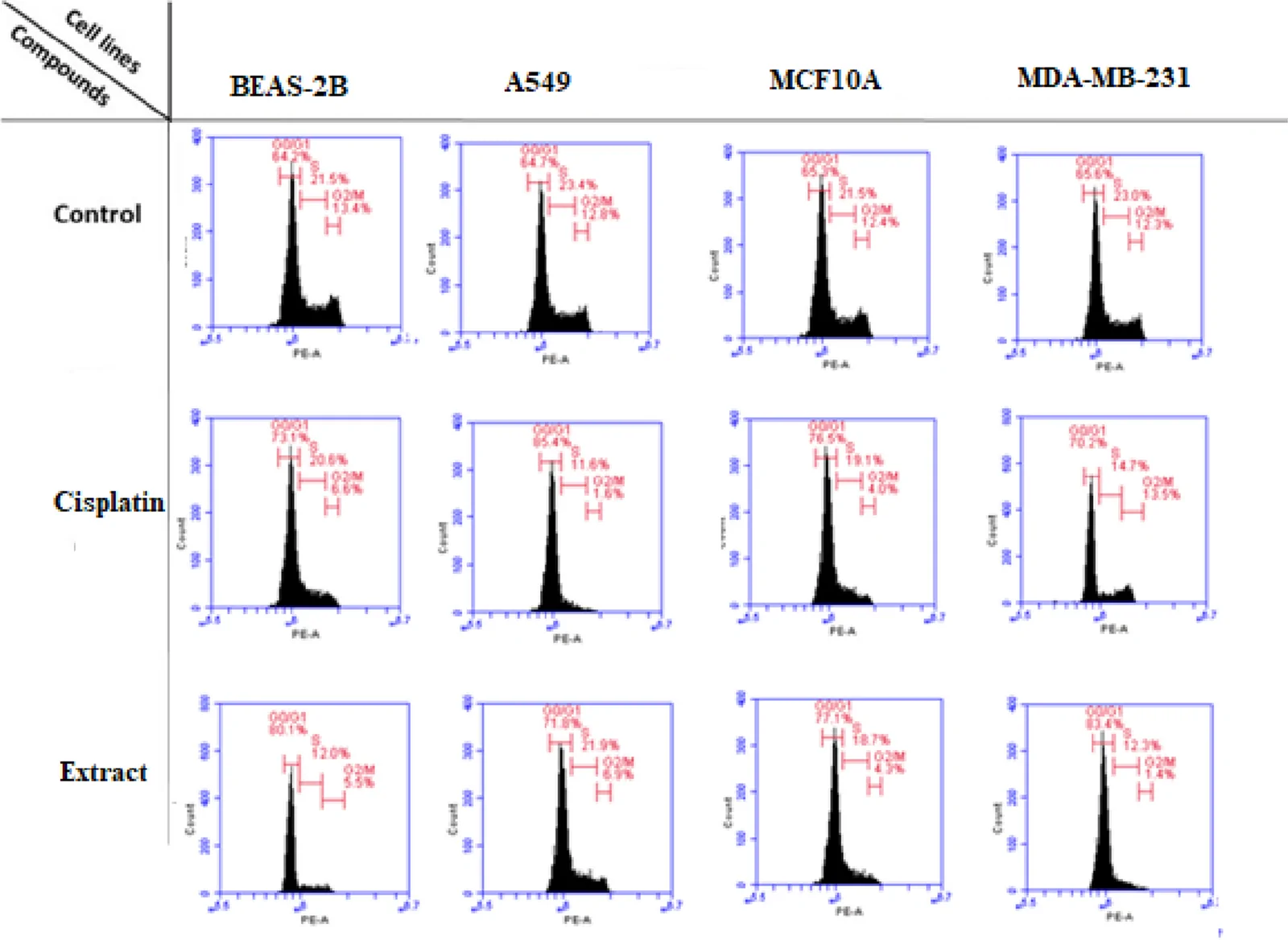
The flow cytometry images of the cell cycle results in BEAS, A549, MCF10A and MDA-MB-231 cells following *E. petrophila* extract and cisplatin treatments

**Fig. 3:**
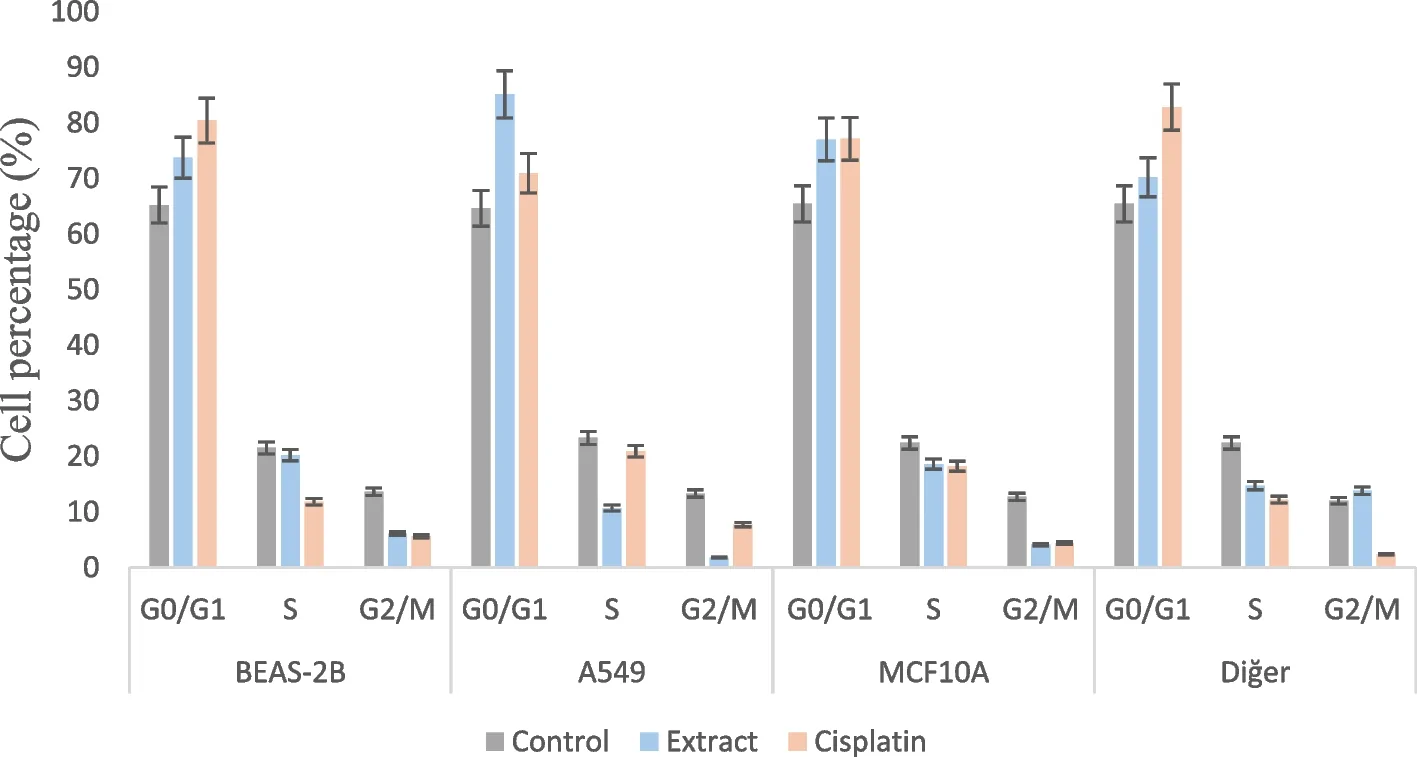
Effects of *E. petrophila* extract on cell cycle distribution in BEAS-2B, A549, MCF10A, and MDA-MB-231 cells. Cells were treated with the extract (25 µg/mL) or cisplatin (20 µg/mL) for 24 h and analyzed via flow cytometry using propidium iodide staining. Data are presented as mean ± SD (*n* = 3). A significant increase in G0/G1 population and a decrease in G2/M phase was observed in treated cancer cells compared to controls (*p* < 0.001)

In this study, the effects of a plant extract and cisplatin treatment on the cell cycle were investigated in BEAS-2B, A549, MCF10A, and MDA-MB-231 cell lines. In BEAS-2B cells, the G0/G1 phase was observed at approximately 65%, 75%, and 80% levels in the control, *E. petrophila*, and cisplatin groups, respectively; the S phase was around 22%, 21%, and 12%; and the G2/M phase was approximately 13%, 5%, and 6%. In A549 cells, the G0/G1 phase was observed at about 66%, 87%, and 71%; the S phase at 24%, 11%, and 12%; and the G2/M phase at approximately 10%, 2%, and 7% for control*, E. petrophila*, and cisplatin groups, respectively. In MCF10A cells, the G0/G1 phase was about 66%, 78%, and 77%; the S phase was around 23%, 18%, and 18%; and the G2/M phase was approximately 12%, 4%, and 4% for the control, *E. petrophila*, and cisplatin groups, respectively. In MDA-MB-231 cells, the G0/G1 phase was approximately 66%, 74%, and 83%; the S phase was around 23%, 15%, and 12%; and the G2/M phase was about 12%, 13%, and 2% in the control, *E. petrophila*, and cisplatin groups, respectively. The flow cytometry images showing the cell cycle results for BEAS, A549, MCF10A and MDA-MB-231 cells after *E. petrophila* extract and cisplatin treatments are presented in Fig. 2. The proliferation percentages of *E. petrophila* extract and cisplatin treatments in these four cell lines are presented in Fig. 3.

### Real-Time PCR results

In the BEAS-2B cell line, the relative mRNA expression levels of TP53, CDKN1A, BRCA1, ATM, CDK4, and CyclinD1 genes were compared across the control, *E. petrophila* extract, and cisplatin treatment groups. The expression of all genes in the control group was set to 1. In the group treated with *E. petrophila*, the relative expression values obtained were TP53: 12.6, CDKN1A: 11.3, BRCA1: 1.8, ATM: 1.6, CDK4: 1.3, and CyclinD1: 1.6. In the cisplatin-treated cells, the values recorded were TP53: 6.14, CDKN1A: 5.74, BRCA1: 1.5, ATM: 0.34, CDK4: 0.7, and CyclinD1: 1.4.

In the A549 cell line, the relative mRNA expression levels of TP53, CDKN1A, BRCA1, ATM, CDK4, and CyclinD1 genes were comparatively evaluated in the control, *E. petrophila* extract, and cisplatin treatment groups. The control group was set as the reference value of 1 for all genes. In A549 cells treated with *E. petrophila*, the relative expression values obtained were TP53: 5.32, CDKN1A: 21.5, BRCA1: 3.2, ATM: 4.2, CDK4: 2.6, and CyclinD1: 2.3. After cisplatin treatment, the recorded values were TP53: 4.23, CDKN1A: 5.03, BRCA1: 1.23, ATM: 4.29, CDK4: 0.64, and CyclinD1: 3.5.

In the RT-PCR analysis conducted in MCF10A cells, the relative expression levels of all genes in the control group were determined to be 1. Following *E. petrophila* treatment, the expression of TP53 increased to 23.6, and CDKN1A expression increased to 15.3 (*p* < 0.05). The relative mRNA expression levels measured were 3.2 for BRCA1, 1.8 for ATM, 2.4 for CDK4, and 2.12 for CyclinD1. After cisplatin treatment, the expression of P53 was recorded at 4.2, CDKN1A expression at 5.03, BRCA1 expression at 1.24, ATM expression at 9.08, CDK4 expression at 0.93, and CyclinD1 expression at 1.04.

**Fig. 4:**
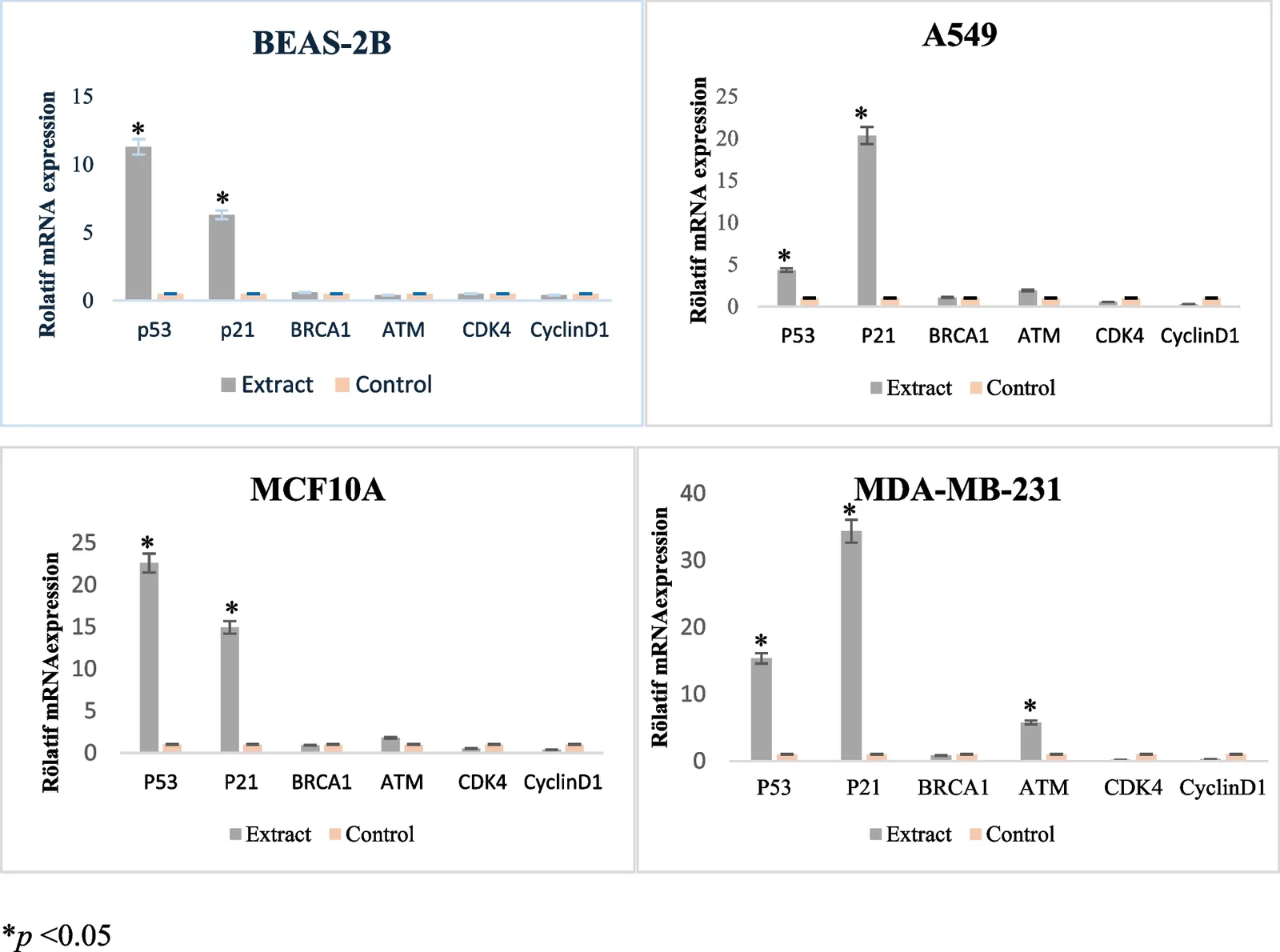
The changes in gene expression following *E. petrophila* extract treatment in BEAS-2B, A549, MCF10A, and MDA-MB-231 cell lines. *p < 0.05

In the MDA-MB-231 cell line, the relative mRNA expression levels of TP53, CDKN1A, BRCA1, ATM, CDK4, and CyclinD1 genes were compared across the control, *E. petrophila* extract, and cisplatin treatment groups. The expression of all genes in the control group was considered as the reference value of 1. In cells treated with *E. petrophila*, the relative mRNA expression levels obtained were TP53: 15.3, CDKN1A: 32.1, BRCA1: 3.4, ATM: 1.8, CDK4: 1.3, and CyclinD1: 1.4. In the cisplatin-treated cells, the recorded values were TP53: 6.24, CDKN1A: 5.03, BRCA1: 0.73, ATM: 0.38, CDK4: 0.68, and CyclinD1: 0.97. The gene expression results after extract treatments in groups are presented in Fig. 4.

### Migration and invasion assessments

**Fig. 5:**
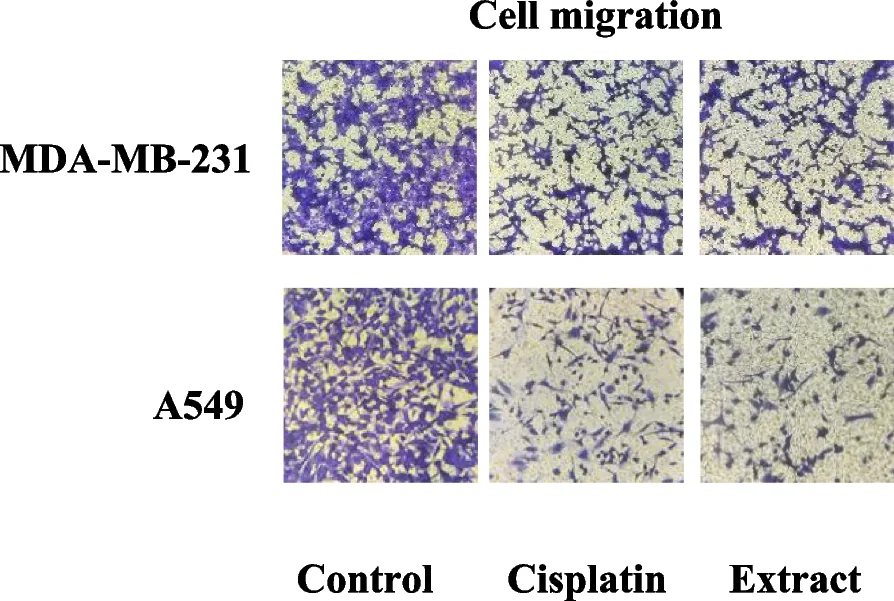
Effects of different treatment groups on cell migration in MDA-MB-231 and A549 cell lines. *p < 0.005

**Fig. 6:**
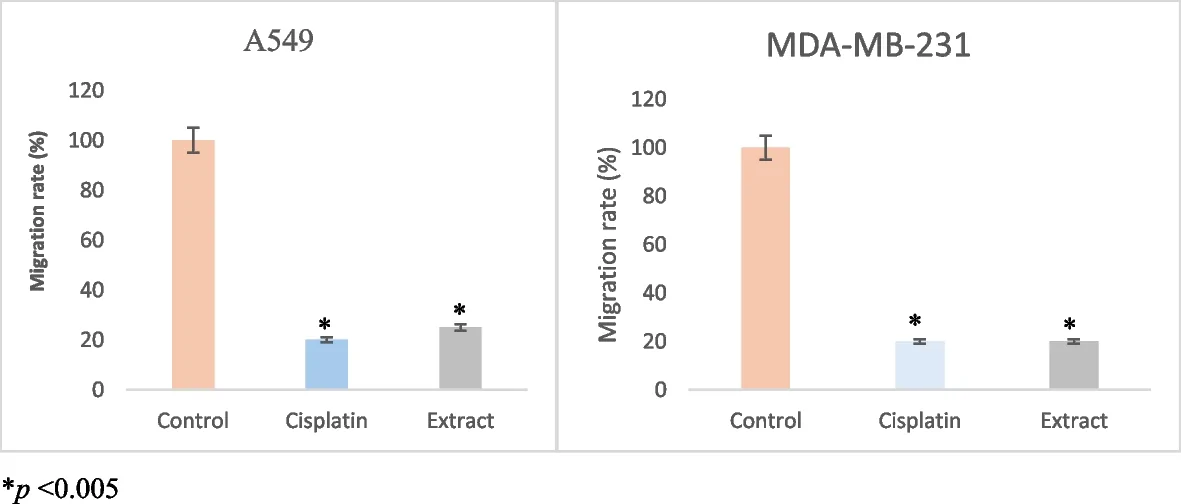
Average number of migrated cells in MDA-MB-231 and A549 cell lines across different treatment groups

Cell migration was evaluated in the A549 and MDA-MB-231 cell lines following treatment with the extract. In the control groups, the migration rate was measured at 100% for both cell lines. After extract application, the migration rate was reduced to approximately 25% in A549 cells and 20% in MDA-MB-231 cells. The microscopic images of the study are shown in Fig. 5, the average cell numbers resulting from migration are presented in Fig. 6, and the intercellular comparisons are provided in Table 7. Because the migration and invasion assays were performed under treatment conditions that may also affect cell viability, these findings should be interpreted as reduced migratory/invasive cell recovery rather than definitive evidence of direct anti-migratory or anti-invasive mechanisms.

**Table 7:** The average cell numbers resulting from migration in the MDA-MB-231 and A549 cell lines after treatment with *E. petrophila* extract and cisplatin are shown

Cell Group	Control	*E. petrophila* extract (% mean ± SD)	Cisplatin (%mean ± SD)	Mean Difference (Δ%)	Significance
A549	100 ± 6.32	25.21 ± 2.23	21.15 ± 2.76	74.79	*p* < *0.05*
MDA-MB- 231	100 ± 5.85	20.45 ± 4.36	20.24 ± 1.25	79.55	*p* < *0.05*

**Fig. 7:**
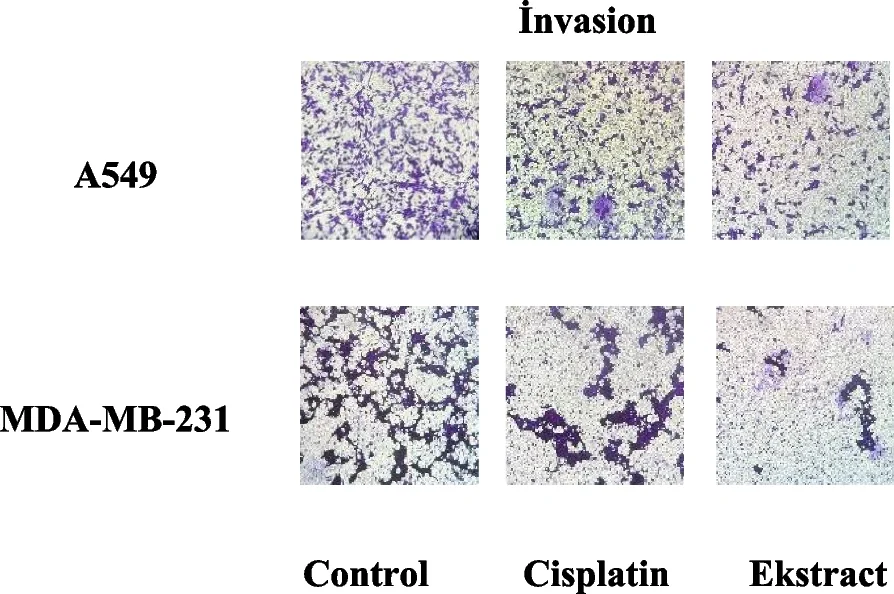
The effects of *E. petrophila* extract and cisplatin treatments on invasion in MDA-MB-231 and A549 cell lines (*p* < 0.05)

**Fig. 8:**
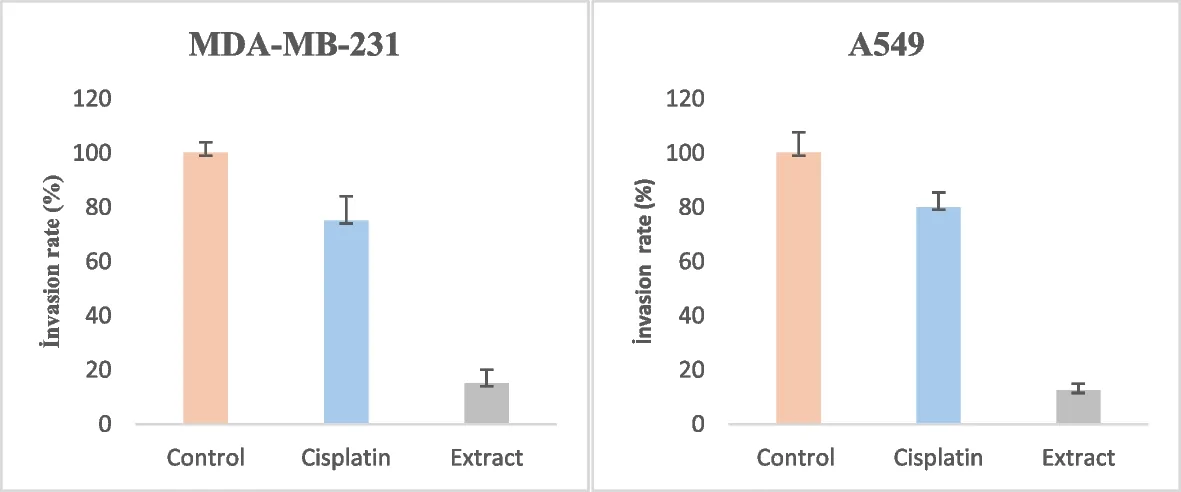
Average number of invaded cells in MDA-MB-231 and A549 cell lines across different treatment groups

Cell invasion was evaluated in the A549 and MDA-MB-231 cell lines following treatment with the extract. In the control groups, the invasion rate was measured at 100% for both cell lines. After extract application, the invasion rate was reduced to approximately 25% in A549 cells and 20% in MDA-MB-231 cells. The microscopic images of the study are shown in Fig. 7, the average cell numbers resulting from migration are presented in Fig. 8, and the intercellular comparisons are provided in Table 8.

**Table 8:** The effects of *E. petrophila* extract and cisplatin treatments on invasion in MDA-MB-231 and A549 cell lines (*p* < 0.05)

Cell Group	Control	*E. petrophila* extract (% mean ± SD)	Cisplatin (% mean ± SD)	Mean Difference (Δ%)	Significance
A549	100 ± 6.58	12.50 ± 2.45	81.54 ± 6.76	87.50	*p* < *0.05*
MDA- MB- 231	100 ± 5.69	15.40 ± 5.56	75.54 ± 7.25	84.60	*p* < *0.05*

*SD* Standard deviation

## Discussion

The present study investigated the phytochemical profile and biological activities of *E. petrophila*, an endemic plant species collected from high altitudes of the Yama Mountain region in Malatya, Turkey. All experimental assays were conducted using ethanol extracts prepared from authenticated herbarium specimens (Herbarium No: 16549–2023, INUH). Taxonomic validation of plant material is critical for scientific traceability and reproducibility, particularly in phytopharmacological studies where misidentification can lead to erroneous interpretations of biological effects [24].

The LC–MS/MS results indicate that *E. petrophila* ethanol extract contains several phenolic acids and flavonoids that may contribute to its observed biological activity. However, because the study tested a crude extract rather than isolated compounds, the contribution of each constituent cannot be determined from the present data.

The presence of hydroxybenzoic acid and salicylic acid in the extract is noteworthy. Both compounds are phenolic acids that have been shown to exhibit strong antioxidant activities [25]. They may contribute to protecting cells from oxidative stress, which is a key factor in the progression of cancer. Oxidative damage can promote tumor growth and metastasis, and the antioxidant activity of these compounds could play a role in mitigating these effects [26]. Hydroxybenzoic acid, in particular, has been associated with the inhibition of cancer cell proliferation, making it an important bioactive component in the extract [27].

The most abundant compound in the extract is gallic acid, which was detected at 13,735.17 ng/mL. Gallic acid is a well-known antioxidant and anti-inflammatory agent with significant anticancer properties [28]. Studies have shown that gallic acid can induce apoptosis in cancer cells and inhibit the growth and migration of various cancer types [29]. This could explain the observed inhibitory effects *of E. petrophila* extract on cancer cell migration and invasion, as demonstrated in previous experiments with A549 and MDA-MB-231 cell lines.

Several flavonoids were also detected in the extract, including apigenin, rutin hydrate, quercetin, isoquercitrin, and kaempferol. Quercetin, in particular, has been extensively studied for its anticancer properties, including its ability to inhibit cell proliferation and migration in various cancer types [30]. Rutin hydrate, which was found at 1068.55 ng/mL, has been shown to reduce the invasion of cancer cells by inhibiting matrix metalloproteinase (MMP) activity, a key enzyme involved in the breakdown of the extracellular matrix (ECM) during metastasis [31]. Isoquercitrin and apigenin also possess potent anticancer and anti-inflammatory effects, modulating pathways involved in cancer progression such as cell cycle arrest and apoptosis [32].

Additionally, ellagic acid and chlorogenic acid were found at substantial levels in the extract, with ellagic acid at 9490.81 ng/mL and chlorogenic acid at 608.96 ng/mL. Both compounds are known for their antioxidant and anticancer effects [33]. Ellagic acid, in particular, has been shown to modulate key signaling pathways such as NF-kB and AP-1, which are involved in inflammation and cancer progression [34].

The coexistence of these compounds may contribute to the observed bioactivity, but synergy was not directly tested. Future studies should use fractionation, compound-depletion, and combination-index approaches to determine whether the extract acts through additive, synergistic, or antagonistic interactions.

The high concentrations of gallic acid, quercetin-related derivatives, rutin, and ellagic acid support the rationale for further mechanistic testing. Nevertheless, the present evidence is insufficient to describe the extract as a therapeutic anticancer agent; it should be described as a candidate extract with preliminary in vitro bioactivity.

The IC₅₀ value of values presented in Table 5 show that *E. petrophila* extract and cisplatin exhibit distinct cytotoxic effects across different cell lines. In BEAS-2B cells (normal lung epithelial cells), cisplatin was more cytotoxic, with an IC₅₀ value of 31.09 ± 0.98 μg/mL, compared *to E. petrophila* extract, which had an IC₅₀ value of 55.52 ± 2.61 μg/mL. This indicates that cisplatin is more effective in reducing the viability of normal lung cells.

In A549 lung cancer cells, *E. petrophila* extract showed stronger cytotoxicity than cisplatin under the tested conditions. This finding is promising but should be interpreted cautiously because it depends on dose, exposure time, assay type, and the lack of broader toxicity data.

For MCF10A cells (normal mammary epithelial cells), cisplatin was slightly more effective, with an IC₅₀ value of 36.66 ± 1.23 μg/mL, compared to *E. petrophila* extract at 43.99 ± 1.92 μg/mL. These findings suggest possible differences in cellular sensitivity to the extract; however, the measurable effects observed in non-tumorigenic cell lines indicate that tumor selectivity cannot be concluded without selectivity index calculations and broader toxicity profiling.

In the MDA-MB-231 (triple-negative breast cancer) cell line, cisplatin was again more cytotoxic, with an IC₅₀ value of 27.31 ± 0.74 μg/mL, compared to *E. petrophila* extract at 62.19 ± 3.10 μg/mL. Although the extract showed differential effects across malignant and non-tumorigenic cell lines, the measurable reduction in viability and increased apoptotic populations observed in BEAS-2B and MCF10A cells indicate that tumor selectivity cannot be inferred from the present data alone.

Flow cytometry analysis demonstrated significant apoptotic induction in A549 (23.5%), followed by moderate effects in MCF10A (14.3%) and BEAS-2B (11.2%) cells. Interestingly, MDA-MB-231 cells showed lower apoptosis (10.7%), indicating possible resistance mechanisms. These results were further substantiated by the upregulation of Tp53 and CDKN1A expression levels, particularly in MCF10A and MDA-MB-231 cells (22.6% and 34.3%, respectively). Although *E. petrophila* extract increased TP53 and CDKN1A (p21) expression and promoted G0/G1 accumulation, the transcriptional response of CDK4 and CCND1 was not uniform across cell lines. In A549 and MCF10A cells, CDK4 and/or CCND1 transcripts were not reduced despite clear evidence of cell-cycle redistribution, suggesting that mRNA expression alone may not reflect the functional inhibition of CyclinD–CDK4/6 complexes. These findings support the possibility that p21-dependent checkpoint control may occur through post-transcriptional or post-translational regulatory mechanisms rather than simple transcriptional repression of CDK4 and Cyclin [35, 36].

On the other hand, *E. petrophila* extract may be associated with apoptotic responses through mechanisms that could potentially involve extrinsic death receptor related and or intrinsic mitochondrial pathways; however, direct activation of these pathways was not demonstrated in the present study and requires confirmation through protein level and functional assays [37]. Flavonoids and phenolic acids found in *E. petrophila* extract, such as quercetin, gallic acid, and ellagic acid, have been shown to upregulate p53 and CDKN1A expression, both of which are involved in cell cycle arrest and apoptosis. It is possible that ROS-related cellular stress may contribute to the apoptotic response induced by *E. petrophila* extract; however, ROS production, mitochondrial dysfunction, cytochrome c release, and caspase-9/caspase-3 activation were not directly demonstrated in this study. Therefore, involvement of the intrinsic mitochondrial apoptotic pathway should be interpreted as a hypothesis requiring further experimental validation [38]. The marked TP53 induction in non-tumorigenic cells highlights the need for genotoxicity evaluation.

The differential apoptotic responses observed among the tested cell lines suggest that *E. petrophila* extract may exert cell line–dependent effects. In A549 cells, the extract was associated with a higher apoptotic rate than cisplatin under the current experimental conditions; however, the mechanistic basis of this response remains uncertain. The potential involvement of multiple apoptotic pathways cannot be concluded without direct evidence of pathway activation, such as caspase activity, mitochondrial dysfunction, death receptor signaling, or DNA damage markers. Conversely, cisplatin induced a stronger apoptotic response in MDA-MB-231 cells, indicating that treatment sensitivity may vary according to cell-specific molecular features. Overall, these findings should be regarded as preliminary evidence of differential treatment responsiveness rather than definitive proof of distinct apoptotic mechanisms.

Exposure to *E. petrophila* extract induced a stress-responsive transcriptional pattern characterized by increased *TP53* and *CDKN1A (p21)* expression in both malignant and non-malignant epithelial cell models. Together with the observed increase in apoptotic cell populations and the accumulation of cells in the G0/G1 phase, these findings are consistent with activation of cell-cycle checkpoint–related responses. Nevertheless, because the present study assessed transcript levels rather than protein activation or pathway dependency, the data should be interpreted as supportive of an association rather than definitive evidence of a p53/p21-mediated mechanism. In addition, the absence of uniform suppression of *CDK4* and *CCND1* across all cell lines suggests that transcriptional responses may not directly reflect functional CDK4/6 inhibition and that post-transcriptional regulation may also be involved [39]. Notably, CDK4 and CCND1 transcripts were not consistently downregulated across all cell lines, suggesting that transcriptional changes alone may not reliably indicate functional CDK4/6 inhibition. Although CDKN1A may potentially contribute to G0/G1 accumulation through post-transcriptional or post-translational regulation of Cyclin D–CDK complexes, this possibility requires confirmation by protein-level analyses and functional CDK activity assays. Furthermore, the pronounced induction of TP53 and CDKN1A in BEAS-2B and MCF10A cells highlights the need to define the extract’s therapeutic window more clearly and to directly assess potential DNA damage using approaches such as γH2AX detection or comet assay. These additional analyses would help determine whether the observed stress response reflects tumor-selective activity or a broader genotoxic effect in both malignant and non-tumorigenic epithelial cells [40]. In the TNBC model MDA-MB-231, interpretation of TP53 upregulation warrants caution given the frequent presence of mutant TP53 in this lineage, raising the possibility of TP53-independent CDKN1A regulation [41].

The obtained data show that both *E. petrophila* extract and cisplatin treatments significantly inhibited migration in MDA-MB-231 and A549 cell lines. In A549 cells, migration rates after treatment were 25.21% ± 2.23 for *E. petrophila* extract and 21.15% ± 2.76 for cisplatin. In MDA-MB-231 cells, migration rates were 20.45% ± 4.36 and 20.24% ± 1.25, respectively, for the same treatments. Compared to the control group, *E. petrophila* extract inhibited migration by 74.79% in A549 cells and 79.55% in MDA-MB-231 cells (*p* < 0.05). These findings indicate that *E. petrophila* extract has a strong potential to inhibit cancer cell migration and, consequently, limit tumor invasion.

The observed inhibition of cell migration may be associated with broader stress-response and cell-cycle regulatory changes induced by *E. petrophila* extract. Although ATM and ATR are key checkpoint kinases involved in the cellular response to DNA damage, the present study did not directly demonstrate DNA damage or activation of ATM/ATR signaling. Therefore, the relationship between migration inhibition and DNA damage response pathways should be considered hypothetical and requires further validation using protein-level and functional assays [42]. Upon activation by DNA damage, ATM/ATR signaling may regulate TP53 and CDKN1A, two important mediators of cell-cycle control. In the present study, *E. petrophila* extract and cisplatin treatments were associated with increased TP53 and CDKN1A mRNA expression, accumulation of cells in the G0/G1 phase, and reduced migratory activity [43].

The stronger reduction in migration observed after *E. petrophila* extract treatment may reflect cell line–specific differences in stress-response, cell-cycle redistribution, or viability-related effects. However, because ATM/p53/p21 pathway activation and functional dependency were not directly demonstrated, this interpretation should remain hypothetical, which is known to regulate cell cycle progression and cell motility [44]. The stronger reduction in migration observed after *E. petrophila* extract treatment may reflect cell line–specific differences in stress-response, cell-cycle redistribution, or viability-related effects. However, because ATM/ATR activation, p53/p21 protein regulation, and pathway dependency were not directly demonstrated, the mechanistic basis of migration inhibition remains uncertain [45, 46].

Although cisplatin also inhibits migration through CDKN1A transcriptional activation and cell cycle arrest, its effect on migration is less potent compared to *E. petrophila* extract. This discrepancy may reflect the different mechanisms by which cisplatin and *E. petrophila* extract exert their effects. Cisplatin primarily induces DNA damage and halts cell cycle progression at the G2/M phase, Although cisplatin and *E. petrophila* extract showed different patterns of migration inhibition, the present data are insufficient to define distinct mechanistic routes for these effects. Future studies should assess DNA damage markers, ATM/ATR activation, p53/p21 protein levels, and pathway inhibition to clarify whether these pathways contribute directly to migration suppression [47].

The results demonstrate that both *E. petrophila* extract and cisplatin treatments significantly inhibited invasion in MDA-MB-231 and A549 cell lines. In the A549 cell line, *E. petrophila* extract treatment reduced invasion to 12.50% ± 2.45, while cisplatin reduced invasion to 81.54% ± 6.76, compared to the control group (100% ± 6.58). Similarly, in the MDA-MB-231 cell line, *E. petrophila* extract reduced invasion to 15.40% ± 5.56, and cisplatin reduced invasion to 75.54% ± 7.25, compared to the control group (100% ± 5.69%). The differences in invasion are substantial, with *E. petrophila* extract reducing invasion by 87.50% (A549) and 84.60% (MDA-MB-231) relative to the control (*p* < 0.05). These findings suggest that *E. petrophila* extract possesses potent anti-invasive properties, surpassing cisplatin in inhibiting cancer cell invasion in both cell lines.

The significant reduction in cell invasion by *E. petrophila* extract correlates with its potential to target key molecular pathways involved in cancer metastasis. One likely mechanism of action involves the inhibition of Epithelial-Mesenchymal Transition (EMT), a key process in cancer cell invasion and metastasis [48]. During EMT, epithelial cells acquire mesenchymal properties that enhance their migratory and invasive capacities [49]*. E. petrophila* extract may interfere with the activation of EMT markers such as N-cadherin, vimentin, and Snail, thus reducing cancer cell invasion [50].

Cisplatin, a well-known chemotherapeutic agent, likely inhibits invasion through its ability to induce DNA damage and activate the TP53 pathway [51]. Activation of TP53 can lead to cell cycle arrest and apoptosis, limiting the ability of cancer cells to invade surrounding tissues [52]. However, in this study, cisplatin showed less potent inhibition of invasion compared to *E. petrophila* extract, which could indicate that cisplatin’s effects are more focused on inducing cell death rather than specifically targeting the molecular pathways that regulate invasion.

Furthermore, Matrix Metalloproteinases (MMPs), particularly MMP-2 and MMP-9, play a critical role in the breakdown of the extracellular matrix (ECM) and enable cancer cells to invade surrounding tissues [53]. The marked reduction in invasion observed after *E. petrophila* extract treatment may be related to changes in invasion-associated cellular processes, potentially including extracellular matrix remodeling. Nevertheless, MMP expression, MMP enzymatic activity, and ECM degradation were not directly evaluated in this study. Therefore, the involvement of MMP-dependent mechanisms remains speculative and should be confirmed in future studies using assays such as gelatin zymography, MMP-2/MMP-9 expression analysis, or ECM degradation assays [54].

The inhibition of cell motility is another crucial aspect of invasion suppression [55]. The marked reduction in invasion suggests that *E. petrophila* extract may influence invasion-associated cellular behavior. However, the present study did not assess EMT markers, MMP activity, cytoskeletal remodeling, or PI3K/Akt and MAPK/ERK signaling. Therefore, the molecular basis of the anti-invasive response remains unclear and should be investigated in future mechanistic studie [56]. By modulating these pathways, *E. petrophila* extract could reduce the ability of cancer cells to migrate and invade. In contrast, cisplatin primarily exerts its effects by inducing DNA damage and activating apoptotic pathways, with less direct impact on the signaling pathways regulating cell motility and invasion.

The marked reduction in invasion suggests that *E. petrophila* extract may influence invasion-associated cellular behavior. However, the present study did not assess EMT markers, MMP enzymatic activity, cytoskeletal remodeling, or PI3K/Akt and MAPK/ERK signaling. Therefore, the molecular basis of the observed anti-invasive response remains unclear and should be investigated in future studies.

## Conclusion

This study provides initial in vitro evidence that *Euphorbia petrophila* ethanol extract exhibits antiproliferative, pro-apoptotic, anti-migratory, and anti-invasive activities in selected breast and lung cancer cell models, with a particularly pronounced response in A549 cells. These effects were associated with increased *TP53* and *CDKN1A* mRNA expression and G0/G1 cell-cycle accumulation, suggesting possible involvement of stress-response and cell-cycle regulatory processes. However, because the present findings are limited to in vitro functional assays and transcript-level analyses, they should be interpreted as preliminary and correlative rather than mechanistically definitive. The measurable effects observed in non-tumorigenic epithelial cells further indicate that he extract showed selective cytotoxicity toward A549 cells compared with BEAS-2B cells; however, selectivity was not observed for MDA-MB-231 compared with MCF10A. Future studies should include selectivity index calculations, extended toxicity profiling, LC–MS/MS method validation, compound-specific analyses, protein-level mechanistic validation, and in vivo models to clarify the pharmacological relevance and safety profile of *E. petrophila* extract.

## Supplementary Information

Supplementary Material 1.

Supplementary Material 2.

Supplementary Material 3.

## Data Availability

All data supporting the findings of this study are available within the paper and its Supplementary Information.

## Abbreviations

ANOVA: Analysis of variance
ATCC: American Type Culture Collection
CDKN1A: Cyclin-dependent kinase inhibitor 1A
DMEM: Dulbecco’s modified Eagle medium
ESI: Electrospray ionization
FBS: Fetal bovine serum
LC–MS/MS: Liquid chromatography–tandem mass spectrometry
MTT: 3-(4,5-Dimethylthiazol-2-yl)-2,5-diphenyltetrazolium bromide
PI: Propidium iodide
RT-qPCR: Quantitative real-time polymerase chain reaction
SD: Standard deviation
SI: Selectivity index
TP53: Tumor protein p53

## References

[1] Paleari L. Personalized assessment for cancer prevention, detection, and treatment. MDPI; 2024. p. 8140. 10.3390/ijms25158140 PMC11311957 39125710

[2] Bray F, et al. Global cancer statistics 2022: GLOBOCAN estimates of incidence and mortality worldwide for 36 cancers in 185 countries. CA Cancer J Clin. 2022;74(3):229–63. 10.3322/caac.21834 38572751

[3] Tsao M. PL01. 05 The new WHO classification of lung tumors. J Thorac Oncol. 2021;16(3):S63. 10.1016/j.jtho.2021.11.003 34808341

[4] Knezevic CE, Clarke W. Cancer chemotherapy: the case for therapeutic drug monitoring. Ther Drug Monit. 2020;42(1):6–19. 10.1097/FTD.0000000000000701 31568180

[5] Islam MR, et al. Colon cancer and colorectal cancer: prevention and treatment by potential natural products. Chem Biol Interact. 2022;368:110170. 10.1016/j.cbi.2022.110170 36202214

[6] Shi Q-W, Su X-H, Kiyota H. Chemical and pharmacological research of the plants in genus *Euphorbia*. Chem Rev. 2008;108(10):4295–327. 10.1021/cr078350s 18817355

[7] Kemboi D, et al. A review of the ethnomedicinal uses, biological activities, and triterpenoids of *Euphorbia* species. Molecules. 2020;25(17):4019. 10.3390/molecules25174019 PMC7504773 32899130

[8] Benjamaa R, et al. *Euphorbia* species latex: a comprehensive review on phytochemistry and biological activities. Front Plant Sci. 2022;13:1008881. 10.3389/fpls.2022.1008881 PMC9583255 36275519

[9] Silva VAO, et al. In vitro screening of cytotoxic activity of euphol from *Euphorbia tirucalli* on a large panel of human cancer‑derived cell lines. Exp Ther Med. 2018;16(2):557–66. 10.3892/etm.2018.6244 PMC6090420 30112023

[10] Wang ZY, et al. Anticancer potential of *Euphorbia helioscopia* L extracts against human cancer cells. Anat Rec (Hoboken). 2012;295(2):223–33. 10.1002/ar.21517 22190452

[11] Cheng F, et al. A natural triterpene derivative from *Euphorbia kansui* inhibits cell proliferation and induces apoptosis against rat intestinal epithelioid cell line in vitro. Int J Mol Sci. 2015;16(8):18956–75. 10.3390/ijms160818956 PMC4581281 26274958

[12] Mishra R, et al. Role of reactive oxygen species and targeted therapy in metastatic melanoma. Cancer Res Front. 2018;4(1):101–30.

[13] Costa JAd, et al. Phytochemical profile, toxicological screening, antitumor activity, and immunomodulatory response of saline extract from *Euphorbia hirta* L. leaves. Molecules. 2025;30(15):3105. 10.3390/molecules30153105 PMC12348506 40807278

[14] Shin SY, et al. *Euphorbia humifusa* Willd exerts inhibition of breast cancer cell invasion and metastasis through inhibition of TNFα-induced MMP-9 expression. BMC Complement Altern Med. 2016;16(1):413. 10.1186/s12906-016-1404-6 PMC5078950 27776550

[15] Amirghofran Z, et al. Tumor cell death via apoptosis and improvement of activated lymphocyte cytokine secretion by extracts from *Euphorbia **hebecarpa* and *Euphorbia **petiolata*. Asian Pac J Cancer Prev. 2019;20(7):1979. 10.31557/APJCP.2019.20.7.1979 PMC6745218 31350954

[16] Glauser G, et al. Ultra-high pressure liquid chromatography–mass spectrometry for plant metabolomics: a systematic comparison of high-resolution quadrupole-time-of-flight and single stage Orbitrap mass spectrometers. J Chromatogr A. 2013;1292:151–9. 10.1016/j.chroma.2012.12.009 23274073

[17] Yang Z, Xiong HR. Culture conditions and types of growth media for mammalian cells. In: Ceccherini-Nelli L, Matteoli B, editors. Biomedical Tissue Culture. IntechOpen. 2012:3–18. 10.5772/52301.

[18] Chan GKY, et al. A simple high-content cell cycle assay reveals frequent discrepancies between cell number and ATP and MTS proliferation assays. PLoS ONE. 2013;8(5):e63583. 10.1371/journal.pone.0063583 PMC3656927 23691072

[19] Güzel MA, Kolaç T, Menevşe İN, et al. Integrated chemical and biological characterization of Hypericum perforatum extract using LC-MS/MS and in vitro functional assays. Sci Rep. 2026;16:6276. 10.1038/s41598-026-36793-8. PMC12905129 41593177

[20] Khan S, et al. Real-time quantitative-PCR (qrt-PCR) using Alleleseqr technology: to assess chimerism in pediatrics patients undergoing hematopoietic stem cell transplantation (HSCT). Biol Blood Marrow Transplant. 2012;18(2):S354.

[21] Nair A, Manohar SM. A flow cytometric journey into cell cycle analysis. Bioanalysis. 2021;13(21):1627–44. 10.4155/bio-2021-0071 34601886

[22] Henry CM, Hollville E, Martin SJ. Measuring apoptosis by microscopy and flow cytometry. Methods. 2013;61(2):90–7. 10.1016/j.ymeth.2013.01.008 23403105

[23] Valster A, et al. Cell migration and invasion assays. Methods. 2005;37(2):208–15. 10.1016/j.ymeth.2005.08.001 16288884

[24] Soejarto DD, Addo EM, Kinghorn AD. The continuing need for taxonomic input in phytochemical research. J Ethnopharmacol. 2025. 10.1016/j.jep.2025.120474. PMC12407247 40876791

[25] Lunić T, Petković M, Rakić M, Lađarević J, Repac J, Božić Nedeljković B, et al. Hydroxybenzoic acid esters as dual antioxidant and anti-neuroinflammatory agents: in silico and in vitro evaluation. In: Proceedings of the 1st International Electronic Conference on Medicinal Chemistry and Pharmaceutics; 1–30 Nov 2025; online. Basel: MDPI; 2025.

[26] Chen D, et al. Targeting oxidative stress-mediated regulated cell death as a vulnerability in cancer. Redox Biol. 2025;84:103686. 10.1016/j.redox.2025.103686 PMC12159232 40424719

[27] Kutraite I, Augustiniene E, Malys N. Hydroxybenzoic acids: microbial metabolism, pathway engineering and products. Biotechnol Adv. 2025;81:108571. 10.1016/j.biotechadv.2025.108571 40154763

[28] Aydoğan A, Kanat Ö. Molecular basis of gallic acid-mediated anticancer activity. In: Akça HŞ, Ünal R, editors. Innovative Applications and Research Methods in Health Sciences. 1st ed. Platanus Publishing; 2025. p. 379–96.

[29] Nasser RM, Elebiary NA, Alomari KB. Gallic acid and its derivatives: a review of their antioxidant properties and applications for fossil fuel preservation. Egypt J Chem. 2025;68(11):277–97. 10.21608/ejchem.2025.357652.11253.

[30] Chen Y, et al. Exploring alkaloids and flavonoids from natural sources: emerging natural agents for inhibiting cervical cancer progression through apoptosis induction, anti-inflammatory effects, and oxidative stress reduction. Pathol Res Pract. 2025;272:156092. 10.1016/j.prp.2025.156092 40596791

[31] Alfawaz M, et al. Therapeutic potential of food-derived rutin phytosome nanoparticles: anti-tumor, antioxidant, and anti-inflammatory activity in Ehrlich ascites carcinoma. Pharmaceuticals (Basel). 2025;18(9):1410. 10.3390/ph18091410 PMC12473802 41011277

[32] Li H, et al. Isoquercitrin attenuates osteoarthritis progression by targeting P53-mediated ferroptosis: a mechanistic study integrating network pharmacology and experimental validation. J Nutr Biochem. 2026. 10.1016/j.jnutbio.2026.110306. 41663037

[33] Huang X, et al. Ellagic acid on milk production performance, blood and milk hormones, antioxidant capacity and fecal microbial communities in lactating Yili mares. Front Microbiol. 2025;16:1656100. 10.3389/fmicb.2025.1656100 PMC12380849 40881293

[34] Eldesoqui M, et al. Ellagic acid protects against gastric ulcer in male rats by antioxidant and anti-inflammatory mechanisms: modulation of NF-κβ/COX-2 and Nrf2/HO-1 pathways. Arch Physiol Biochem. 2025;131(6):1045–60. 10.1080/13813455.2025.2529295 40620128

[35] Obeng E. Apoptosis (programmed cell death) and its signals-A review. Braz J Biol. 2020;81(4):1133–43. 10.1590/1519-6984.228437 33111928

[36] Rane CK, Minden A. P21 activated kinase signaling in cancer. In: Seminars in cancer biology. Elsevier; 2019. 10.1016/j.semcancer.2018.01.006 29330094

[37] Mosadegh M, Noori Goodarzi N, Erfani Y. A comprehensive insight into apoptosis: molecular mechanisms, signaling pathways, and modulating therapeutics. Cancer Investig. 2025;43(1):33–58. 10.1080/07357907.2024.2445528 39760426

[38] Çınar İ, Akyel S, Dincer B. Protective effects of engeletin on doxorubicin-induced cardiotoxicity via NF-κB/iNOS and Cyt-c/CASP-3 signaling pathways. Cukurova Med J. 2025;50(3):872–82.

[39] Pandey SN, et al. ATM and p53 in aging and cancer: a double-edged sword in genomic integrity. Biogerontology. 2025;26(3):102. 10.1007/s10522-025-10249-4 40323544

[40] Chiang J-C, et al. Lipoylation inhibition enhances radiation control of lung cancer by suppressing homologous recombination DNA damage repair. Sci Adv. 2025;11(11):eadt1241. 10.1126/sciadv.adt1241 PMC11900879 40073141

[41] Thangavelu L, et al. Targeting the p53–p21 axis in liver cancer: linking cellular senescence to tumor suppression and progression. Pathol Res Pract. 2024;263:155652. 10.1016/j.prp.2024.155652 39437639

[42] Karakostis K, et al. The DNA damage sensor ATM kinase interacts with the p53 mRNA and guides the DNA damage response pathway. Mol Cancer. 2024;23(1):21. 10.1186/s12943-024-01933-z PMC10804554 38263180

[43] Engeland K. Cell cycle regulation: p53–p21-RB signaling. Cell Death Differ. 2022;29(5):946–60. 10.1038/s41418-022-00988-z PMC9090780 35361964

[44] Kozyrska K, et al. P53 directs leader cell behavior, migration, and clearance during epithelial repair. Science. 2022;375(6581):eabl8876.10.1126/science.abl887635143293

[45] Li X, et al. G1/S arrest: a key mechanism of cellular aging and replicative senescence. Biogerontology. 2026;27(1):40. 10.1007/s10522-025-10378-w 41543765

[46] Fu T, et al. p21 promotes gemcitabine tolerance in A549 cells by inhibiting DNA damage and altering the cell cycle. Oncol Lett. 2023;26(5):471. 10.3892/ol.2023.14059 PMC10551858 37809050

[47] Yu J-B, et al. Repeated administration of cisplatin transforms kidney fibroblasts through G2/M arrest and cellular senescence. Cells. 2022;11(21):3472. 10.3390/cells11213472 PMC9655665 36359868

[48] Liaghat M, et al. The impact of epithelial-mesenchymal transition (EMT) induced by metabolic processes and intracellular signaling pathways on chemo-resistance, metastasis, and recurrence in solid tumors. Cell Commun Signal. 2024;22(1):575. 10.1186/s12964-024-01957-4 PMC11610171 39623377

[49] Alvarez CL, Troncoso MF, Espelt MV. Extracellular ATP and adenosine in tumor microenvironment: roles in epithelial–mesenchymal transition, cell migration, and invasion. J Cell Physiol. 2022;237(1):389–400. 10.1002/jcp.30580 34514618

[50] Tabatabaee A, et al. Targeting vimentin: a multifaceted approach to combatting cancer metastasis and drug resistance. Cancer Metastasis Rev. 2024;43(1):363–77. 10.1007/s10555-023-10154-7 38012357

[51] Liu S, et al. Androgen receptor suppresses lung cancer invasion and increases cisplatin response via decreasing TPD52 expression. Int J Biol Sci. 2023;19(12):3709. 10.7150/ijbs.84577 PMC10411467 37564195

[52] Liu Y, et al. p53-regulated non-apoptotic cell death pathways and their relevance in cancer and other diseases. Nat Rev Mol Cell Biol. 2025;26(8):600–14. 10.1038/s41580-025-00842-3 PMC13014097 40204927

[53] Sabir U, Gu H, Zhang DW. Extracellular matrix turnover: phytochemicals target and modulate the dual role of matrix metalloproteinases (MMPs) in liver fibrosis. Phytother Res. 2023;37(11):4932–62. 10.1002/ptr.7959 37461256

[54] Akhlaghipour I, Moghbeli M. Matrix metalloproteinases as the critical regulators of cisplatin response and tumor cell invasion. Eur J Pharmacol. 2024;982:176966. 10.1016/j.ejphar.2024.176966 39216742

[55] Contreras PS, et al. Differential contribution of TFE3 isoforms to cell motility and invasion. EMBO Rep. 2025;27(2):471. 10.1038/s44319-025-00659-3 PMC12852735 41361692

[56] Raghavan S, Pattabiraman PP. Rho GTPase and Rho kinase signaling in tissue contractility and intraocular pressure regulation. Front Biosci. 2026. 10.31083/FBL44589. 41609085

