# Supplementary material for: LC–MS/MS-based phytochemical profiling and preliminary in vitro anticancer-related bioactivity of Euphorbia petrophila ethanol extract
Source: BMC Complement Med Ther. 2026 Jul 23;26:309. doi: 10.1186/s12906-026-05470-8 (PMC13678952; doi:10.1186/s12906-026-05470-8)
Supplement: Supplementary file 1 [file 12906_2026_5470_MOESM1_ESM.docx]

**Appendix 1**

**Figure 1.** LC-MS/MS chromatogram of the *E. petrophila* extract.

This chromatogram presents the chromatographic separation of phenolic acid and flavonoid secondary metabolites within the analyzed extract, distributed according to their retention times (RT). An examination of the analytical profile reveals the successful separation and formation of distinct, well-resolved peaks for specific compounds at their respective retention times. These include quinic acid, gallic acid, gentisic acid, salicylic acid, quercetin 3-rutinoside 7-glucoside, and naringenin. Notably, the high peak intensity observed in the initial minute of the run (attributed to quinic acid), combined with the clear chromatographic distribution across the subsequent minutes, corroborates the structural diversity of the major phytochemicals in the plant extract and demonstrates the high-resolution capacity of the applied analytical method.
