# Supplementary material for: LC–MS/MS-based phytochemical profiling and preliminary in vitro anticancer-related bioactivity of Euphorbia petrophila ethanol extract
Source: BMC Complement Med Ther. 2026 Jul 23;26:309. doi: 10.1186/s12906-026-05470-8 (PMC13678952; doi:10.1186/s12906-026-05470-8)
Supplement: Supplementary file 2 [file 12906_2026_5470_MOESM2_ESM.docx]

Supplementary Material

**Figure 9**. LC-MS/MS Chromatogram
